# The complete chloroplast genome of *Aquilaria malaccensis* Lam. (Thymelaeaceae), an important and threatened agarwood-producing tree species

**DOI:** 10.1080/23802359.2018.1519382

**Published:** 2018-10-29

**Authors:** Shiou Yih Lee, Wei Lun Ng, Rozi Mohamed, Razak Terhem

**Affiliations:** a Forest Biotechnology Laboratory Department of Forest Management Faculty of Forestry, Universiti Putra Malaysia, Serdang, Selangor, Malaysia;; b State Key Laboratory of Biocontrol and Guangdong Provincial Key Laboratory of Plant Resources School of Life Sciences, Sun Yat-sen University, Guangzhou, Guangdong, China;; c China-ASEAN College of Marine Sciences, Xiamen University Malaysia, Sepang, Selangor, Malaysia;; d Department of Forest Management Faculty of Forestry, Universiti Putra Malaysia, Serdang, Selangor, Malaysia

**Keywords:** Conservation, comparative genomics, phylogenomics, plant DNA barcoding, Thymelaeaceae

## Abstract

Known for its valuable agarwood, *Aquilaria malaccensis* Lam. is an evergreen tropical forest tree species endemic to the Indo-malesian region. Indiscriminate damaging and harvesting of the trees in the wild have resulted in it being listed in the CITES Appendix II for controlled trade and in the IUCN Red List as ‘Vulnerable (VU)’. In this study, the complete chloroplast genome of *A. malaccensis* was assembled using data from high-throughput Illumina sequencing. The chloroplast genome was 174,832 bp in size, which included two inverted repeat regions of 42,091 bp each, separated by a large single copy region of 87,302 bp and a small single copy region of 3,348 bp. A total of 139 genes were predicted, including 39 tRNA, 8 rRNA, and 92 protein-coding genes. Phylogenetic analysis placed *A. malaccensis* within the family Thymelaeaceae. The chloroplast genome sequence of *A. malaccensis* offers a useful resource for future studies on the taxonomy and conservation of the threatened *Aquilaria* trees.

*Aquilaria malaccensis* Lam. (Thymelaeaceae), is native to the Indo-malesian region and is the most widely distributed species of its genus (Lee and Mohamed 2016). In response to pathogenic infection via external wounds, an *Aquilaria* tree secretes resin, which hardens and turns into agarwood (Mohamed et al. 2010), a valuable ingredient for traditional medicines, perfumery, and religious uses. As natural agarwood is rare, intentional wounding and indiscriminate felling of wild *Aquilaria* trees in search of agarwood have resulted in the decline of natural *Aquilaria* populations over the years, prompting conservationist and trade enforcers to swiftly decide on protecting these trees in the wild (Barden et al. 2000). Since 1998, *A. malaccensis* has been given the status of ‘Vulnerable (VU)’ by the IUCN Red List (IUCN 2017), and also became the first agarwood-producing species under the tribe Aquilarieae to make it into Appendix II of CITES in 2005 (UNEP-WCMC 2015). Due to commercial value, studies that focus on the agarwood often overshadow studies on the *Aquilaria* trees that produce them, allowing taxonomical issues within the genus to persist. In this study, we characterized the complete cp genome sequence of *A. malaccensis* as a resource for future studies on the taxonomy of *Aquilaria*.

Genomic DNA was extracted from fresh leaves of *A. malaccensis* grown in the greenhouse of the Faculty of Forestry, Universiti Putra Malaysia (UPM), originally obtained from a natural population in Pahang, Malaysia. A voucher specimen (FBL01001) is deposited at the Forest Biotechnology Laboratory in the Faculty of Forestry, UPM. A genomic library with an insert size of 300 bp was prepared using a TruSeq DNA Sample Prep Kit (Illumina, CA, USA) and sequenced on an Illumina HiSeq X Ten platform. Approximately 8 Gb of raw data was generated through pair-end 150 bp sequencing. After removal of adapter sequences, raw reads were fed into the assembly pipeline of NOVOPlasty (Dierckxsens et al. 2017) with the *rbc*L sequence of *Aquilaria yunnanensis* (GenBank accession KR528756) as the seed sequence. The assembled cp genome was annotated using the online annotation tool DOGMA (Wyman et al. 2004) and further checked manually by comparison against the *A. yunnanensis* cp genome (MG656407).

The complete cp genome of *A. malaccensis* (GenBank accession MH286934) was 174,832 bp in length, consisting of a pair of inverted repeat regions of 42,091 bp each, a large single copy region of 87,302 bp, and a small single copy region of 3,348 bp. A total of 139 genes were annotated, including 92 protein-coding genes, 39 transfer RNA genes, and 8 ribosomal RNA genes. The overall GC content of the cp genome was 36.71%.

Phylogenetic analysis was conducted on an alignment consisting of chloroplast genome sequences of six species in the order Malvales, including four species from the family Thymelaeaceae (*A. malaccensis*, *A. sinensis*, *A. yunnanensis*, and *Daphne kiusiana*), two species from the family Malvaceae (*Gossypium arboretum*, and *Theobroma cacao*), and one outgroup species, *Eucalyptus grandis* from the family Myrtaceae. A maximum likelihood tree constructed with RAxML (Stamatakis 2014), implemented in Geneious ver. 10.1 (http://www.geneious.com, Kearse et al. 2012) placed *A. malaccensis* together with other species of *Aquilaria* (Figure 1).

**Figure 1.**
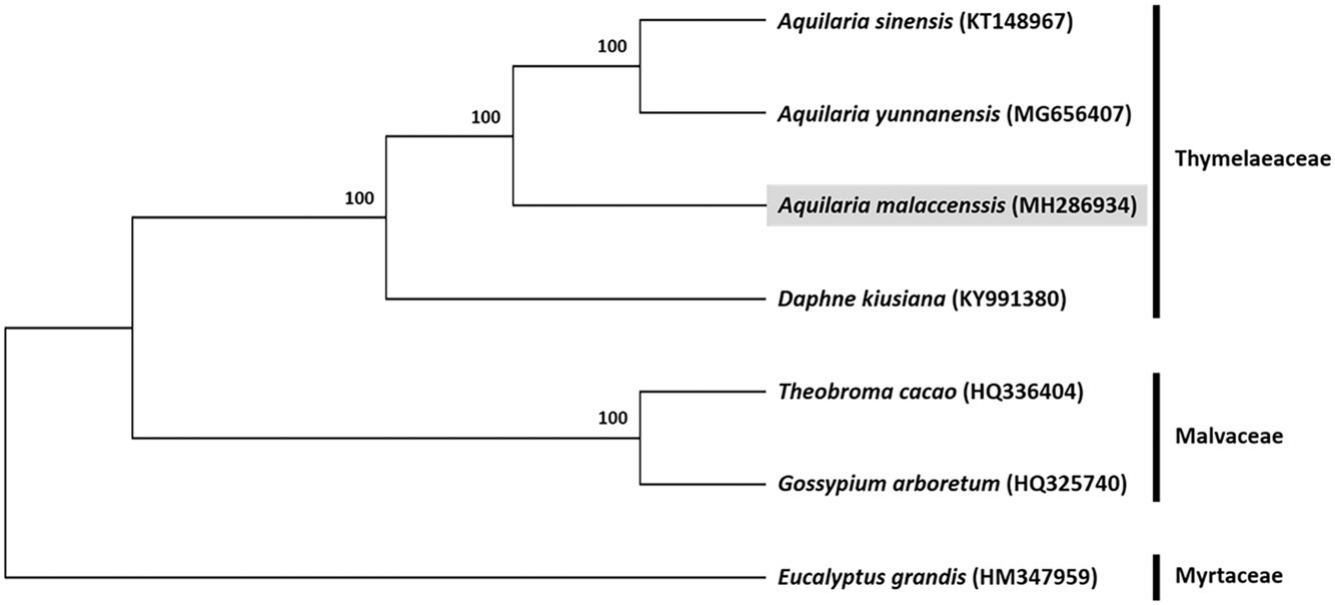
Maximum likelihood tree based on the complete chloroplast genome sequences of six species from the order Malvales, with *Eucalyptus grandis* (order Myrtales, family Myrtaceae) as the outgroup. Shown next to the nodes are bootstrap support values based on 1000 replicates.
